# Dealloying-Derived Nanoporous Cu_6_Sn_5_ Alloy as Stable Anode Materials for Lithium-Ion Batteries

**DOI:** 10.3390/ma14154348

**Published:** 2021-08-03

**Authors:** Chi Zhang, Zheng Wang, Yu Cui, Xuyao Niu, Mei Chen, Ping Liang, Junhao Liu, Runjun Liu, Jingcong Li, Xin He

**Affiliations:** School of Applied Physics and Materials, Wuyi University, Jiangmen 529020, China; wangzh1104@163.com (Z.W.); CuiYu2468@163.com (Y.C.); nxy13007149948@163.com (X.N.); chenmei116@126.com (M.C.); ping_liang@126.com (P.L.); wyuljh@163.com (J.L.); lrj3118004629@163.com (R.L.); lijingcong1026@163.com (J.L.)

**Keywords:** dealloying, nanoporous, Cu6Sn5 alloy, lithium-ion battery

## Abstract

The volume expansion during Li ion insertion/extraction remains an obstacle for the application of Sn-based anode in lithium ion-batteries. Herein, the nanoporous (np) Cu_6_Sn_5_ alloy and Cu_6_Sn_5_/Sn composite were applied as a lithium-ion battery anode. The as-dealloyed np-Cu_6_Sn_5_ has an ultrafine ligament size of 40 nm and a high BET-specific area of 15.9 m^2^ g^−1^. The anode shows an initial discharge capacity as high as 1200 mA h g^−1^, and it remains a capacity of higher than 600 mA h g^−1^ for the initial five cycles at 0.1 A g^−1^. After 100 cycles, the anode maintains a stable capacity higher than 200 mA h g^−1^ for at least 350 cycles, with outstanding Coulombic efficiency. The ex situ XRD patterns reveal the reverse phase transformation between Cu_6_Sn_5_ and Li_2_CuSn. The Cu_6_Sn_5_/Sn composite presents a similar cycling performance with a slightly inferior rate performance compared to np-Cu_6_Sn_5_. The study demonstrates that dealloyed nanoporous Cu_6_Sn_5_ alloy could be a promising candidate for lithium-ion batteries.

## 1. Introduction

The development of portable electronic devices urgently requires lithium-ion batteries (LIBs) with a high capacity and long lifespan [1,2,3]. Currently, extensive efforts have been devoted to developing alternative anode materials, e.g., metal oxide, alloys and group IVA elements, to boost the capacity of LIBs [1,4,5]. Micro-pillar splitting and micro-cantilever bending have been proposed to evaluate the mechanical behaviors of materials at the micro-scale, and micro-pillar splitting is commonly employed to analyze the fracture toughness of battery electrode materials and solid state electrolyte [6]. Tin (Sn) is regarded as one of the promising anode substitutes due to its high theoretical gravimetric capacity (994 mA h g^−1^ for Li_22_Sn_5_), low cost, great safety and low working potential window (0.3–0.6 V) [7,8,9]. However, Sn suffers from violent irreversible capacity loss and disintegration due to its Li-driven volume expansion (up to 259%) during lithiation and de-lithiation cycling, which impedes its application in commercial LIBs [7,10].

Several approaches have been proposed to overcome the volume expansion issue. One is to reduce the size of the Sn-based materials, i.e., construction of nanostructures. For instance, a 3D nanocomposite consisted of 3D structured Sn scaffold and a hollow Sn sphere was fabricated as the LIBs anode [11]. The anode exhibited a capacity of 1700 mA h cm^−3^ for over 200 cycles at 0.5 C. The high cycling stability could be attributed to the 3D nanostructured design that accommodated the volume expansion during charge/discharge [11]. Another effective approach is to construct Sn/carbon or Sn/metal composite, in which the carbon substrate or metal can act as the buffer to bear the volume change of Sn. Wang’s group designed a mesoporous carbon/Sn anode for Li-ion and Na-ion batteries, in which the mesoporous carbon was proposed to accommodate the volume change of Sn nanoparticles during ion insertion and extraction [12]. A Sn/graphite anode presented improved capacity retention with only 0.7% loss per cycle [13]. Nanocable-like Sn-core/carbon-sheath anode materials have been fabricated by chemical vapor deposition, delivering a specific capacity of 630 mA h g^−1^ after 100 cycles at a current density of 100 mA g^−1^ [14]. In addition, graphene-confined Sn nanosheets and 3D hierarchical SnO_2_/graphene frameworks were designed, showing enhanced lithium storage capability [15,16]. Liu et al. reported a 3D nanocomposite including both a 3D structured Sn scaffold and a hollow Sn sphere within each cavity where all the free Sn surfaces are coated with carbon, showing a high volumetric capacity of ∼1700 mA h cm^−3^ over 200 cycles at 0.5 C, and a capacity greater than 1200 mA h cm^−3^ at 10 C [11]. Combining Sn with metals could form intermetallic compounds and the metal could also act as the buffer to accommodate the volume expansion [1,17]. A Ni_3_Sn_2_ microcage showed stable capacity of 534 mA h g^−1^ after 1000 cycles at the current density of 1 C [17]. Single-crystalline FeSn_2_ nanospheres with uniform small particle size have been synthesized, showing better electrochemical performances than other nano-spherical intermetallic compounds such as Cu_6_Sn_5_, CoSn_3_ and Ni_3_Sn_4_, due to its crystal structure [18]. In addition, Sn-Co alloy nanoparticles encapsulated in a porous 3D graphene network was also applied as the LIB anode [19].

In addition to the above-mentioned materials, the most studied Sn-based materials are Cu-Sn alloys, as copper owns high conductivity and elasticity, and Cu_6_Sn_5_ possesses the high theoretical capacity of 605 mA h g^−1^ [20,21,22,23,24,25]. Several approaches have been applied to fabricate Cu_6_Sn_5_ nanoparticles, film, or nanowires [20,26,27,28,29]. A solution route was used to synthesize dendrite Cu_6_Sn_5_ powers [27]. A facile one-step electrodepositing method could also form Cu_6_Sn_5_ alloy on the Cu foil, and the alloy electrode showed a discharge capacity of 462 mA h g^−1^ [30]. A core-shell Cu@Cu_6_Sn_5_ nanowire was synthesized via an electrodeposition process, and the anode showed excellent rate performance even at high current density of 20 C [31]. Recently, dealloying, a powerful method to fabricate nanoporous metals, was applied to fabricate Cu_6_Sn_5_ nanostructure for fabrication LIB anode [22,25,28]. Liu et al. dealloyed Cu_17_Sn_7_Al_76_ alloy in a 1 M NaOH to obtain a 3D nanoporous Cu-Sn electrode. The electrode presents a capacity of 566 mA h g^−1^ at a current density of 1670 mA g^−1^ [25]. The study found that the nanopores facilitated the penetration of Li^+^ ions. A nanoporous Cu_6_Sn_5_/Cu composite was generated via a similar method by dealloying Al_10_Cu_3_Sn alloy [22,32]. The hierarchical porous structure promoted the mass transport and accommodated the volume change; thus, the as-dealloyed composite displayed enhanced stability and rate performance [22].

In our previous report, we found that by changing Cu and Sn ratio in the Mg-Cu-Sn alloy, Cu-Sn alloys or composite with different phase compositions can be achieved via dealloying [28]. Herein, in this study, we applied the as-dealloyed Cu_6_Sn_5_ alloy and Cu_6_Sn_5_/Sn composite as the LIB anode. The as-dealloyed anode was found to have excellent cycling stability and rate performance due to the nanoporous structure. Moreover, the anode material has good thermal stability due to the formation of intermetallic Cu_6_Sn_5_, favoring the cycling. The ex situ XRD proved the reverse phase transformation between Cu_6_Sn_5_ and Li_2_CuSn during charge/discharge process.

## 2. Experimental

### 2.1. Synthesis of Nanoporous Cu_6_Sn_5_ Alloy and Cu_6_Sn_5_/Sn Composite

Mg_67_Cu_18_Sn_15_ and Mg_66_Cu_10.2_Sn_23.8_ (at.%) precursor ribbons were prepared from rapid solidification. Pure Mg, Cu and Sn metal blocks (purity: 99.9 wt.%) were melted in an alumina crucible using a resistance furnace (KYKY Technology Co., Ltd., Shenyang, China) under the protection of covering agent at about 800 °C. The melt liquid was cast into an alloy rod in a module. The obtained alloy rod was cut into ingots and remelted at 800 °C using a high-frequency induction furnace (KYKY Technology Co., Ltd., Shenyang, China) in a quartz tube with a pinhole below. The alloy melt was blasted onto a copper roller spinning at 1000 revolutions per minute from the pinhole by an Ar blow. The precursor ribbons obtained were collected for dealloying. The dealloying of Mg-Cu-Sn precursor ribbons was performed in a 1 wt.% tartaric acid (TA) at room temperature until no bubbles emerged. The as-dealloyed ribbons were rinsed with deionized water and dehydrated alcohol for further characterizations and applications.

### 2.2. Microstructural Characterization

The microstructures of the as-dealloyed samples were investigated with scanning electron microscope (SEM, LEO 1530P, LEO Electron Microscopy Ltd., Oberkochen, Germany) and transmission electron microscope (TEM, Philips CM 20, FEI Company, Hillsboro, OR, USA). The crystalline nature was probed using high-resolution TEM (HRTEM, FEI Tecnai G2, Thermo Fisher Scientific Inc., Waltham, MA, USA) and selected-area electron diffraction (SAED, FEI Tecnai G2, Thermo Fisher Scientific Inc., Waltham, MA, USA). Nitrogen adsorption/desorption isotherms were measured with a surface area and porosity analyzer (Gold APP V-Sorb 2800, Gold APP Instruments, Beijing, China) at 77 K. Specific surface area was determined using the Brunauer–Emmett–Teller (BET) method. Pore size distribution was calculated from the adsorption branch by the Barrett–Joyner–Halenda (BJH) method using the corrected form of Kelvin equation. The ex situ XRD patterns of the discharged/charged samples were determined by a XD-3 diffractometer (Beijing Purkinje, Beijing Purkinje General Instrument Co., Ltd., Beijing, China) equipped with Cu-Ka radiation as described before [33,34]. The batteries were cycled to the required voltage and then opened for the ex situ XRD testing. A dimethyl carbonate (DMC) solution was used to remove the lithium salt of the cycled electrodes before testing.

### 2.3. Electrochemical Measurement

The as-dealloyed samples were ball-milled to fine powders with zirconia beads at a rotation speed of 300 rpm for 3 h. The Cu_6_Sn_5_ or Cu_6_Sn_5_/Sn powders (80 wt.%) were ground with acetylene black (Super-P, 10 wt.%) and polyvinylidene fluoride (PVDF) binder (10 wt.%) in N-methyl-2-pyrrolidinone (NMP) solvent to make a slurry. The slurry was coated onto Cu foil and further dried at 80 °C for 12 h under vacuum. The coated Cu foil was punched into disk shape of 12 mm in diameter, with the low mass loading of around 0.5 mg/cm^2^. The as-prepared foil was used as the working electrode. A Li foil was used as both the reference electrode and counter electrode and a polypropylene (PP) film (Celgard 2325) was utilized as the separator. The electrolyte was made by mixing 1.0 M LiPF_6_ with ethylene carbonate (EC) and DMC (1:1 by volume). The CR2032-type coin cells were assembled in an argon-filled glove box (Mikrouna Co. Ltd., Shanghai, China), with oxygen and moisture levels below 0.1 ppm. The galvanostatic discharging/charging tests were conducted at various current densities at the voltage window between 0.01 and 2.00 V (vs. Li^+^/Li) using a LANDCT2001A test system. Cyclic voltammetry (CV) was conducted at a scan rate of 0.1 mV s^−1^ between 0.01 and 2.00 V (vs. Li^+^/Li) on an electrochemical workstation (CHI 660E).

## 3. Results and Discussions

**Figure 1:**
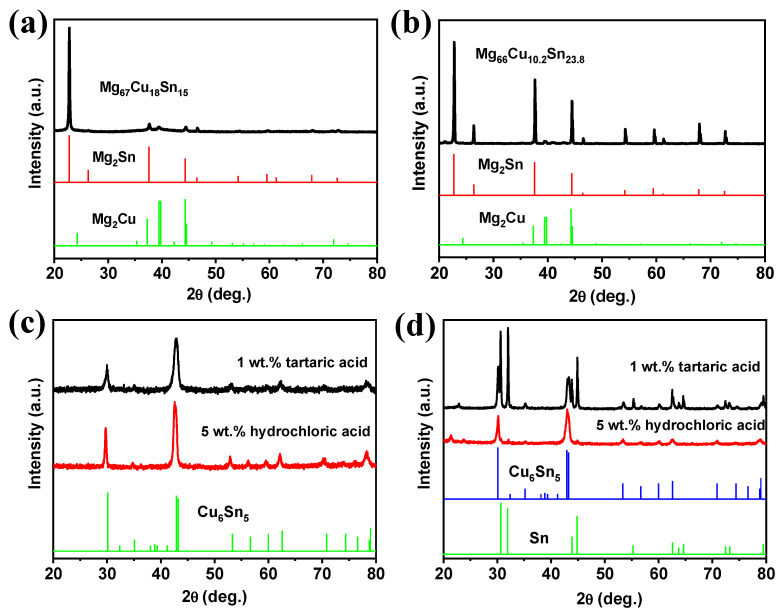
XRD patterns of (**a**) Mg_67_Cu_18_Sn_15_ and (**b**) Mg_66_Cu_10.2_Sn_23.8_ precursors; (**c**) dealloyed Cu_6_Sn_5_ and (**d**) dealloyed Cu_6_Sn_5_/Sn composite. Reproduced from Ref. [28] with permission from the Royal Society of Chemistry.

**Figure 2:**
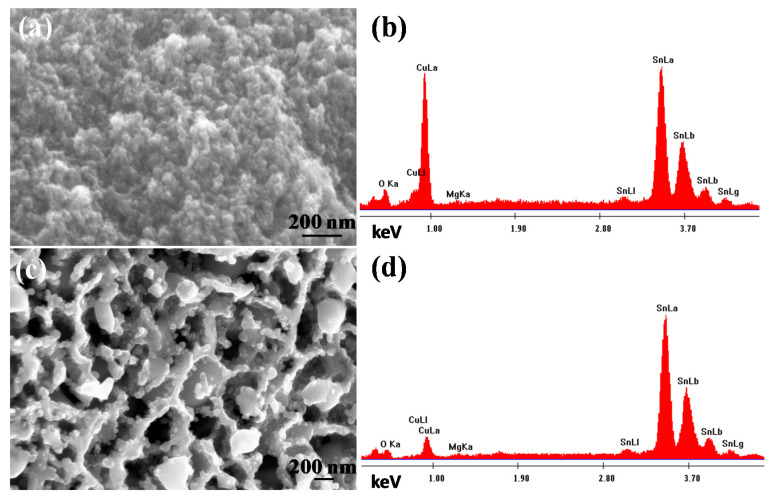
The SEM images and corresponding EDS spectra of (**a**,**b**) Mg_67_Cu_18_Sn_15_ and (**c**,**d**) Mg_66_Cu_10.2_Sn_23.8_ after dealloying in 1 wt.% TA.

**Figure 3:**
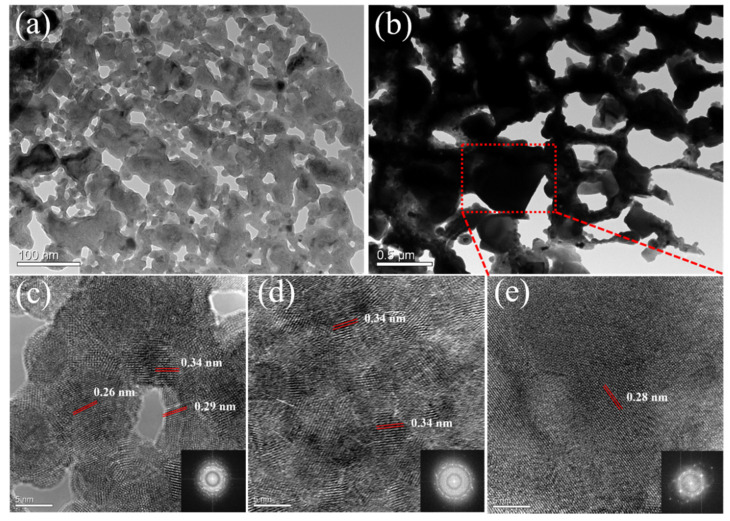
TEM images of dealloyed (**a**) Mg_67_Cu_18_Sn_15_ and (**b**) Mg_66_Cu_10.2_Sn_23.8_; HRTEM of dealloyed (**c**) Mg_67_Cu_18_Sn_15_, (**d**) ligament domain and (**e**) particle domain of Mg_66_Cu_10.2_Sn_23.8_. Insets of (**c**–**e**) are the corresponding FFT patterns.

The phase compositions of the precursors and dealloyed samples were confirmed by XRD in Figure 1. As exhibited in our previously reported study [28], the starting precursors Mg_67_Cu_18_Sn_15_ (Figure 1a) and Mg_66_Cu_10.2_Sn_23.8_ (Figure 1b) both consist of Mg_2_Sn (PDF # 65-2997) and Mg_2_Cu (PDF # 65-5460) phases. Only a minor amount of Mg_2_Cu is present, which indicates that substantial Cu enters the lattice of Mg_2_Sn to form Mg_2_(Sn,Cu) phase. The dealloying of Mg_67_Cu_18_Sn_15_ (Figure 1c) and Mg_66_Cu_10.2_Sn_23.8_ (Figure 1d) in 1 wt.% TA resulted in the formation of np-Cu_6_Sn_5_ (PDF # 65-2303) alloy and Cu_6_Sn_5_/Sn (PDF # 65-2303/PDF # 65-0296) composites, respectively, as described in ref [28]. It has been discussed that the formation of Cu_6_Sn_5_ involved the selective dissolution of Mg and co-diffusion of Cu and Sn [28]. The phase formation process was different from the dealloying of Al-Cu-Sn alloys, of which heating was required to form Cu-Sn alloys [22,35]. The difference in precursor type not only determines the dealloying process, but also has an effect on the microstructure of dealloyed alloys. As shown in Figure 2a and Figure S1, the as-dealloyed Cu_6_Sn_5_ derived from Mg_67_Cu_18_Sn_15_ has ultra-fine bi-continuous ligament-channel morphology. EDS spectra confirms the co-existence of Cu and Sn (Figure 2b). Magnified TEM image shows that the average pore size is below 40 nm (Figure 3a), which is comparable to that from dealloying of Al_10_Cu_3_Sn in a 20 wt.% NaOH solution [22]. When increasing the content of Sn to 23.8 at.%, large skeletons with particles attached instead of nanoporous ligament-channel structure was formed (Figure 2c or Figure 3b and Figure S1). Combined with XRD results in ref [28], the skeletons should be Cu_6_Sn_5_ while the particles are composed of Sn. EDS spectra in Figure 2d shows higher relative Sn contents in the composite, corresponding to higher Sn ratio in the precursor. HRTEM in Figure 3c shows well-aligned lattices corresponding to as-dealloyed Cu_6_Sn_5_ alloy. The lattice distance indicates that the ligaments are identified to Cu_6_Sn_5_. Moreover, the corresponding FFT pattern (inset of Figure 3c) verifies the nanocrystalline nature of Cu_6_Sn_5_ ligaments. Figure 3d,e exhibit the HRTEM images and corresponding FFT patterns of as-dealloyed Mg_66_Cu_10.2_Sn_23.8_ precursor. The spacing distances of lattice fringes corresponds to the (021) crystal plane (0.34 nm) of Cu_6_Sn_5_ (Figure 3d) and (101) plane (0.28 nm) of Sn (Figure 3e), respectively.

**Figure 4:**
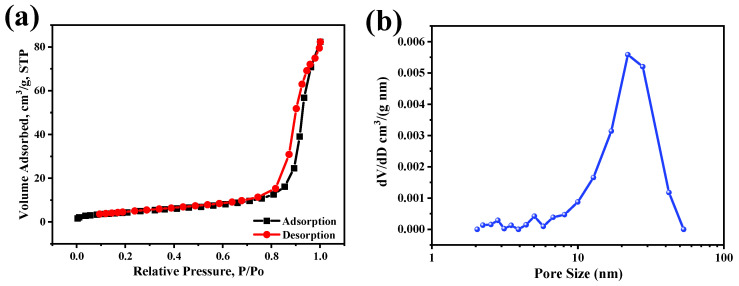
(**a**) N_2_ adsorption/desorption isotherms of the as-dealloyed nanoporous Cu_6_Sn_5_. (**b**) The pore size distribution.

The specific surface areas and pore distributions of as-dealloyed np-Cu_6_Sn_5_ are determined by N_2_ adsorption-desorption method. Figure 4a presents the N_2_ adsorption–desorption isotherms, which corresponds well to the type IV curve with a H3 hysteresis loop, indicating the formation of mesoporosity [36]. The BET-specific surface area is calculated as 15.9 m^2^ g^−1^, which is comparable to np-Cu_6_Sn_5_/Cu composite from the dealloying of Al_10_Cu_3_Sn [22]. The pore size distribution derived from the adsorption branch of the isotherm using the BJH model. The result (Figure 4b) shows that the pore size distributes in the range of 10–40 nm, in accordance with the TEM observation. The relatively high specific surface areas and robust ligament-channel structure might facilitate the diffusion of Li ions and alleviate the volume expansion during the charge/discharge process.

**Figure 5:**
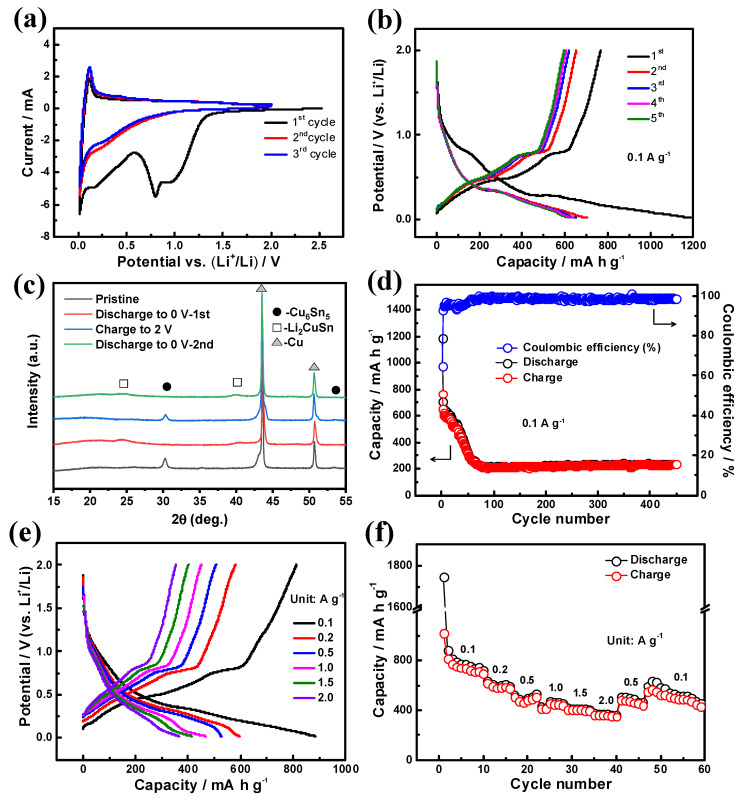
(**a**) CVs at a scan rate of 0.1 mV s^−1^ between 0.01 and 2.5 V (vs. Li^+^/Li). (**b**) The initial five charge/discharge profiles of as-dealloyed Cu_6_Sn_5_ anode with the current density of 0.1 A g^−1^. (**c**) Ex situ XRD patterns collected at various states. (**d**) Cycling performance of the Cu_6_Sn_5_ at current density of 0.1 A g^−1^. (**e**) The charge–discharge voltage profiles of the Cu_6_Sn_5_ alloy electrode at various current densities from 0. 1 A g^−1^ to 2.0 A g^−1^. (**f**) Rate performance at various current densities from 0. 1 A g^−1^ to 2.0 A g^−1^.

CVs at a scan rate of 0.1 mV s^−1^ between 0.01 and 2.5 V (vs. Li^+^/Li) are shown in Figure 5a. In the first cathodic process, np-Cu_6_Sn_5_ anode shows a peak at 0.77 V (vs. Li^+^/Li), due to the irreversible reactions in the initial cycle [22]. The irreversible reactions might be attributed to the formation of solid-electrolyte interface (SEI). The CV peaks start to overlap from the second cycle, indicating that the anode becomes stable after the first cycle (Figure 5a). The performance of as-dealloyed Cu_6_Sn_5_ anode was evaluated using galvanostatic discharge–charge cycling. Figure 5b exhibits the initial five charge/discharge curves between 0.01 V (vs. Li^+^/Li) and 2.0 V (vs. Li^+^/Li) of the dealloyed Cu_6_Sn_5_ anode at the current density of 0.1 A g^−1^. For the first cycle, the discharge process shows a plateau at around 0.25 and 0.77 V (vs. Li^+^/Li), corresponding to the CV curve in Figure 5a. The discharge processes from the second cycle show obvious potential plateau at around 0.3 V (vs. Li^+^/Li), in correspondence with the slight anodic peak from CV curves. The charge processes have plateaus at around 0.5 and 0.75 V (vs. Li^+^/Li), which were also found in other Cu_6_Sn_5_-based materials [21,22,23,24,26]. To further study the phase transition during the charge/discharge process, the ex situ XRD analysis was performed. The pristine np-Cu_6_Sn_5_ clearly shows the peaks indexed to Cu_6_Sn_5_ phase (PDF # 65-2303) (Figure 5c). After fully discharging to 0 V for the first cycle, Cu_6_Sn_5_ phase disappears, and Li_2_CuSn phase (PDF # 65-5125) emerges at around 24.5 °C and 40.6 °C. The XRD pattern corresponding to the fully charged state of the second cycle confirms the transition from Li_2_CuSn to Cu_6_Sn_5_ again. Furthermore, the second fully discharged process proves the reversible conversion from Cu_6_Sn_5_ to Li_2_CuSn. The ex situ XRD patterns indicate the reversible conversion of Cu_6_Sn_5_ to Li_2_CuSn during the charge/discharge process. Figure 5d shows the cycling performance of np-Cu_6_Sn_5_ alloy at the current density of 0.1 A g^−1^. The initial discharge capacity is as high as 700 mA h g^−^^1^, slightly higher than that from electrodeposited Cu_6_Sn_5_ alloy film [26]. The higher capacity could be attributed to the nanoporous ligament-channel structure that facilities the diffusion of electrolyte and the reduced Li-ions insertion pathway into the Cu_6_Sn_5_ ligaments [8,9]. The capacity can be maintained higher than 500 mA h g^−1^ for at least 20 cycles and keep at ~200 mA h g^−1^ for the following 400 cycles with the Coulombic efficiency of nearly 100%. The residual stress of electrodes is related to the performance and stability of energy generation devices such as LIBs here, and np-Cu_6_Sn_5_ with bi-continuous ligament-channel structure own strain relaxation, favoring the cyclability of LIBs [37]. The decay of capacity is induced by the alloy aggregation and collapse of porous structure during cycling, and the stability of np-Cu_6_Sn_5_ anode is expected to be improved by adjusting the constituent of alloy precursors and dealloying conditions in the future. The np-Cu_6_Sn_5_ anode also presents excellent rate performance, as illustrated in Figure 5e,f. At the current density of 0.1 A g^−1^, the capacity is ~900 mA h g^−1^ for the first cycle and maintains at higher than 700 mA h g^−1^ for 10 cycles. With the current density increasing to 2.0 A g^−1^, the capacity gradually decreases to ~380 mA h g^−1^. The anode recovers to a capacity of around 600 mA h g^−1^ when the current density is reversed to 0.1 A g^−1^. The results demonstrate that the nanoporous structure gives an excellent rate performance to the Cu_6_Sn_5_ anode, even after experiencing the high current density of 2.0 A g^−1^. The anode was also performed under 0.5 A g^−1^ to investigate its possible application under a higher current density. As shown in Figure S2, after 100 cycles, the anode can maintain its capacity higher than 150 mA h g^−1^ for at least 1000 cycles, demonstrating excellent stability.

**Figure 6:**
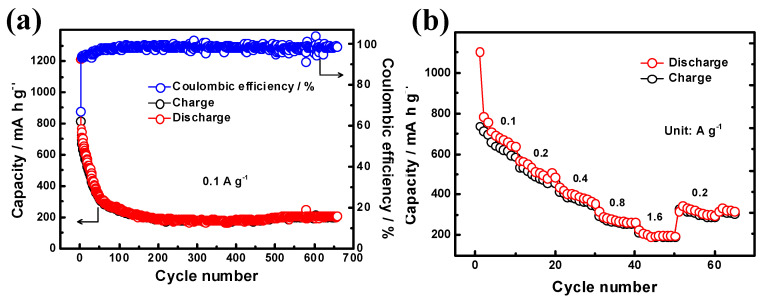
(**a**) Cycling performance of the Cu_16.2_Sn_23.8_ at current density of 0.1 A g^−1^. (**b**) Rate performance at various current densities from 0. 1 A g^−1^ to 1.6 A g^−1^.

The charge/discharge cycling performance of Cu_6_Sn_5_/Sn composite was also tested. The initial charge capacity is higher than 800 mA h g^−1^, better than that of Cu_6_Sn_5_, suggesting the contribution of Sn in the composite (Figure 6a). The capacity can be kept at around 200 mA h g^−1^ for at least 550 cycles, comparable to that of np-Cu_6_Sn_5_. At a higher current density of 0.5 A g^−1^, the capacity could maintain 150 mA h g^−1^ (Figure S3). The Cu_6_Sn_5_/Sn composite also shows an inferior rate performance, as shown in Figure 6b. When the current density was reversed to 0.2 A g^−1^, the capacity could only maintain around 60% of its initial capacity at 0.2 A g^−1^. The inferior cycling performance might be attribute to the Sn particles with large volume expansion during charge/discharge process [1].

## 4. Conclusions

In summary, nanoporous Cu_6_Sn_5_ alloy and Cu_6_Sn_5_/Sn composite were prepared via dealloying and their performance as lithium-ion battery anodes were tested. The dealloyed np-Cu_6_Sn_5_ presented an ultrafine ligament-channel structure with ligament size below 40 nm and BET specific area of 15.9 m^2^ g^−1^. The 3D nanoporous structure and high specific area endowed the anode with high lithium-ion battery performance. The anode displayed a capacity of higher than 600 mA h g^−1^ for the initial five cycles at 0.1 A g^−1^. It also had excellent cycling performance for at least 350 cycles at 0.1 A g^−1^, with a reversible capacity of higher than 200 mA h g^−1^. The ex situ XRD patterns reveal the reverse phase transformation between Cu_6_Sn_5_ and Li_2_CuSn. Similar performance was obtained from Cu_6_Sn_5_/Sn composite. This study provided a practice for fabricating highly stable Sn-based anode in lithium-ion batteries.

## Data Availability

Not applicable.

## Supplementary Materials

The following are available online at https://www.mdpi.com/article/10.3390/ma14154348/s1, Figure S1: SEM image of the dealloyed (a) Mg_67_Cu_18_Sn_15_ and (b) Mg_66_Cu_10.2_Sn_23.8_ alloy, Figure S2: Cycling performance of the Cu_6_Sn_5_ at current density of 0.5 A g^−1^, Figure S3: Cycling performance of the Cu_6_Sn_5_/Sn at current density of 0.5 A g^−1^.
