# Supplementary material for: Dealloying-Derived Nanoporous Cu6Sn5 Alloy as Stable Anode Materials for Lithium-Ion Batteries
Source: Materials (Basel). 2021 Aug 3;14(15):4348. doi: 10.3390/ma14154348 (PMC8346966; doi:10.3390/ma14154348)
Supplement: Supplementary file 1 [file materials-14-04348-s001.zip › materials-1311766-supplementary.pdf]

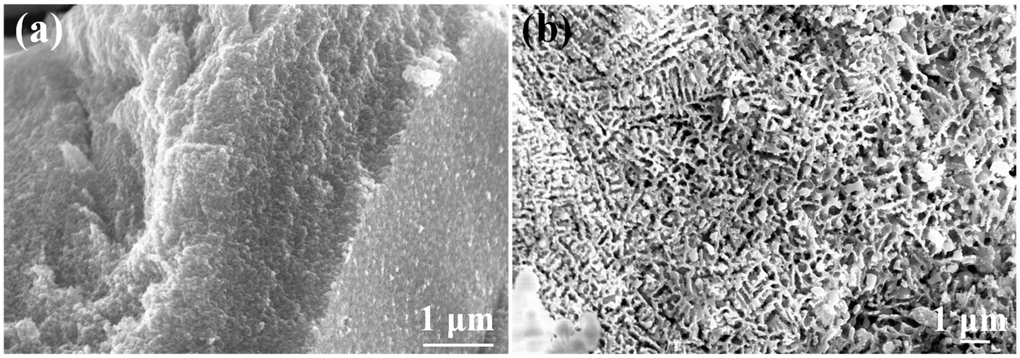

Figure S1. SEM image of the dealloyed (a) $Mg_{67}Cu_{18}Sn_{15}$ and (b) $Mg_{66}Cu_{10.2}Sn_{23.8}$ alloy.

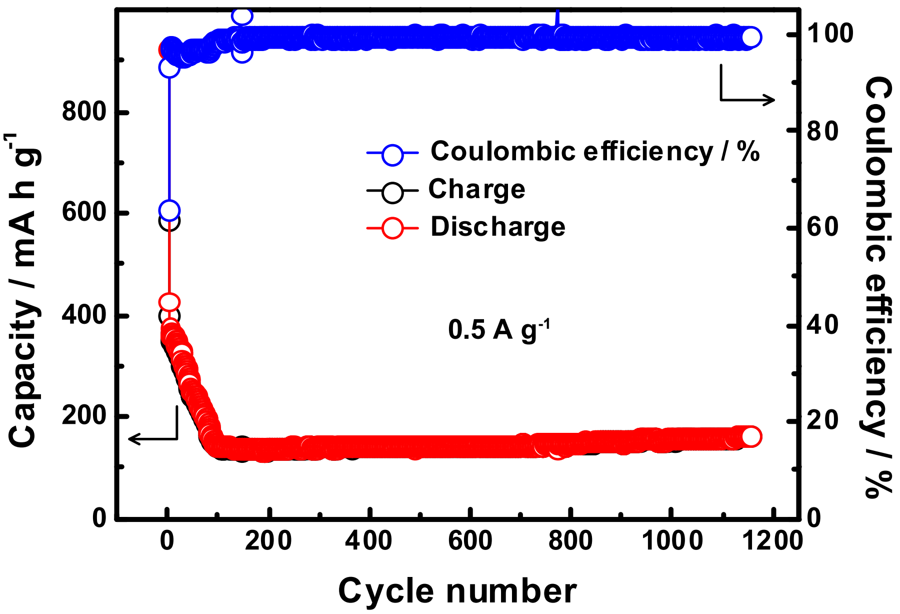

Figure S2. Cycling performance of the Cu_6Sn_5 at current density of 0.5 A g^{-1}.

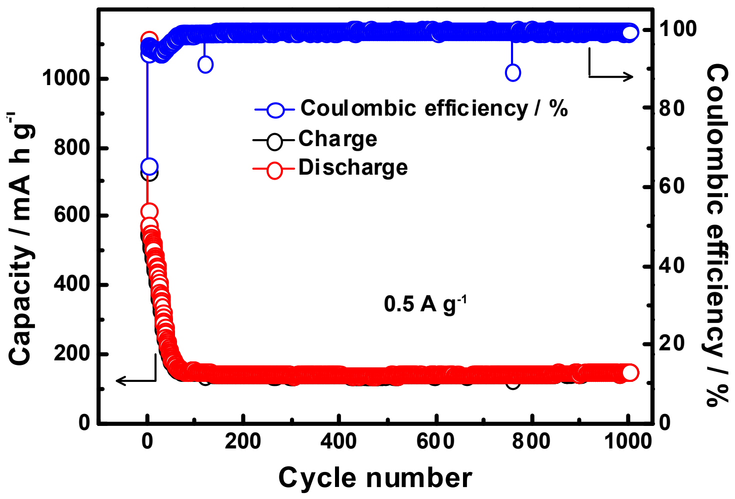

Figure S3. Cycling performance of the Cu_6Sn_5/Sn at current density of 0.5 A g^{-1}.
